# A Whiff of Sulfur: One Wind a Day Keeps the Doctor Away

**DOI:** 10.3390/antiox11061036

**Published:** 2022-05-24

**Authors:** Eduard Tiganescu, Markus Alexander Lämmermann, Yannick Ney, Ahmad Yaman Abdin, Muhammad Jawad Nasim, Claus Jacob

**Affiliations:** Division of Bioorganic Chemistry, School of Pharmacy, Saarland University, D-66123 Saarbruecken, Germany; s9edtiga@stud.uni-saarland.de (E.T.); s9malaem@stud.uni-saarland.de (M.A.L.); yannick.ney@uni-saarland.de (Y.N.); yaman.abdin@uni-saarland.de (A.Y.A.)

**Keywords:** allicin, allyl methyl sulfide (AMS), broccoli, cellular thiolstat, durian, garlic, Reactive Sulfur Species (RSS), sulforaphane

## Abstract

Reactive Sulfur Species (RSS), such as allicin from garlic or sulforaphane from broccoli, are fre-quently associated with biological activities and possible health benefits in animals and humans. Among these Organic Sulfur Compounds (OSCs) found in many plants and fungi, the Volatile Sulfur Compounds (VSCs) feature prominently, not only because of their often-pungent smell, but also because they are able to access places which solids and solutions cannot reach that easily. Indeed, inorganic RSS such as hydrogen sulfide (H_2_S) and sulfur dioxide (SO_2_) can be used to lit-erally fumigate entire rooms and areas. Similarly, metabolites of garlic, such as allyl methyl sulfide (AMS), are formed metabolically in humans in lower concentrations and reach the airways from inside the body as part of one’s breath. Curiously, H_2_S is also formed in the gastrointestinal tract by gut bacteria, and the question of if and for which purpose this gas then crosses the barriers and enters the body is indeed a delicate matter for equally delicate studies. In any case, nature is surprisingly rich in such VSCs, as fruits (for instance, the infamous durian) demonstrate, and therefore these VSCs represent a promising group of compounds for further studies.

## 1. Introduction

Folk medicine has long been interested in plants and fungi rich in sulfur [1,2,3]. During the centuries and across the globe, it has ascribed several potential health benefits to the active ingredients contained in plants such as garlic, onions, mustard, and broccoli, and fungi such as Shiitake [2,4,5,6]. Indeed, at closer inspection, one finds many popular proverbs, for instance across Germany, which link garlic and onions to a healthy digestion and cardiovascular benefits. In Bavaria, home to the fairytale caste Neuschwanstein, for instance, there is an old saying that “if the arse is singing the heart is swinging”, whereas in the Saarland, home to the acclaimed Saarland Hurricanes (Canes) football team, the advice is “to have sunshine in the heart and onions in the belly, then you can breathe freely and smelly” (and not only via the lungs). Although some of this may sound rather offensive initially—just as the battle-cry of the Canes (Let’s go offense, let’s go)—we shall return to some of the possible health benefits associated with such (smelly) metabolites, for instance in the cardiovascular system, in the following sections.

Indeed, the natural products containing sulfur are rather special [7]. Different scientific disciplines have divided such sulfur compounds into specific groups—for instance, the Organic Sulfur Compounds (OSCs) in a more chemical context and the Reactive Sulfur Species (RSS) in a more biological one. Among these OSCs/RSS, the Volatile Sulfur Compounds (VSCs) stand out as they tend to combine high reactivity and hence biological activity with a unique air-bound bioavailability [8,9,10]. No doubt, there are quite a few anecdotes circulating within the scientific community about allicin from garlic fumigating entire incubators and thence affecting cell-based experiments in every corner of the instrument [11,12,13].

In this article, we shall therefore consider different activities and roles ascribed to volatile RSS. We will start with the more prominent activities of H_2_S as a gaseous transmitter in the human body on par with nitric oxide (^●^NO) and the uses of SO_2_ as fumigant and then turn to some of the more exotic and less(er) studied actions of natural substances, such as allicin and its metabolites.

## 2. Inorganic VSCs

In the periodic table, sulfur is found together with oxygen, selenium, tellurium, and polonium in Group 16, elements often referred to as the chalk generating chalcogens. Unlike most other elements, sulfur occurs in many different oxidation states ranging from −2 to +6, and indeed its inorganic chemistry alone may fill entire bookshelves and is quite colorful as far as reactivities and biological activities are concerned. As an appetizer for further reading, we would like to highlight topics such as *S*-thiolation and *O*-sulfation of proteins and enzymes, which may be considered as processes similar to phosphorylation. There is also a barrage of—less(er) studied—inorganic RSS, which contain highly reactive substances, such as hypothiocyanite (OSCN^−^) and thiocyanates (SCN^−^), and have been investigated recently by colleagues such as Michael T. Ashby [14,15,16].

The most prominent and indeed also simple volatile inorganic RSS are H_2_S (melting point −85.49 °C and boiling point −60.33 °C, at atmospheric pressure), with sulfur in its reduced form (oxidation state −2), and SO_2_ (melting point −75.5 °C, boiling point −10.05 °C) with sulfur in oxidized form (oxidation state +4). Please note that SO_3_ with sulfur in oxidation state +6 is not that volatile at room temperature (melting point 16.90 °C, boiling point 44.50 °C).

### 2.1. Hydrogen Sulfide (H_2_S)

Hydrogen sulfide may affect animals and humans via quite a few avenues and in quite a few ways. Firstly, H_2_S occurs in our environment, for instance near volcanos and in mineral waters, and as such may be inhaled and also taken as a bath or orally [17,18]. H_2_S is lethal at higher concentrations exceeding 500–1000 ppm (formally corresponding to 14.7–29.4 mM), whilst at concentrations of 10–500 ppm (0.3–14.7 mM), it affects the respiratory system and symptoms of exposure range from rhinitis to acute respiratory failure [19]. H_2_S is often considered beneficial to human health at lower concentrations and dissolved in water. Bad Nenndorf located in the district of Schaumburg in the State of Lower Saxony in Germany is representative of such a natural mineral sulfur spring with a total sulfur content of 137 ppm, including an H_2_S content ranging from 4–10 ppm (100–300 µM) [20]. Such sulfur-rich water has been employed extensively for the treatment of various types of rheumatic diseases, including degenerative, inflammatory, and soft tissue rheumatism (e.g., fibromyalgia), and also against inflammatory skin diseases, such as eczema and neurodermatitis [20]. Similarly, the Varna basin mineral water in the northern Black Sea region in Bulgaria, which among other sulfur species also contains dissolved H_2_S and soluble sulfides in concentrations ranging from 1.7 to 2.8 ppm (49.9–82.1 µM), has been reported to improve total glutathione and total thiol levels in healthy volunteers with increased expression of γ-glutamyl-cysteinyl ligase (GCL) and soluble intercellular adhesion molecule-1 (sICAM-1) genes [21,22].

Notably, H_2_S is not only of volcanic origin. It is also formed from organic sources, such as decomposing organic materials, and therefore poses a considerable danger in and around old-fashioned cesspits, where concentrations may reach up to 800 ppm in the air (23.5 mM) [23]. If, how, and where such organic sources of H_2_S have or are currently being used for healthy bathing has so far escaped our attention.

Talking about cesspits, H_2_S is also formed in the gastrointestinal tract of most animals and humans by the action of the gut microbiota. In this case, concentrations of H_2_S may reach up to 0.3–3.4 mM in the gut, which is quite impressive considering that H_2_S is already biologically active in micromolar (10–30 μM) concentrations [24,25,26]. In addition, whilst most of the H_2_S formed during digestion may escape like the Saarland Hurricanes on a high note through the backdoor, some of it may also cross the gut-blood barrier and exert its activity inside the body [27,28].

Notably, the production of H_2_S in gut involves microbes belonging to the family of dissimilatory sulfate reducers, such as *Desulfovibrio* and *Bilophila* species. H_2_S is therefore primarily produced in the colon by reduction and not via cleavage of amino acids as in mammalian cells. The concentrations of H_2_S produced by the gut bacteria range from 0.3 to 3.4 mM [29]. Regardless of the mode of production, H_2_S subsequently finds its way into the bloodstream with serum concentrations normally ranging from 34.0 to 36.4 µM in healthy individuals [29,30].

In the gut itself, H_2_S may have both a harmful and a beneficial impact on local microbiota depending upon its concentration. The higher concentrations (high micromolar to millimolar) may lead to the disruption of mucus and inflammation, whilst lower, i.e., nano- and low micromolar concentrations, have been reported to actually stabilize mucus layers, protect biofilm from fragmentation and encourage adherence of the microbiota biofilm to the epithelium, prohibit the escape of invasive pathobionts, and resolve inflammation and tissue injury [31,32,33].

**Figure 1:**
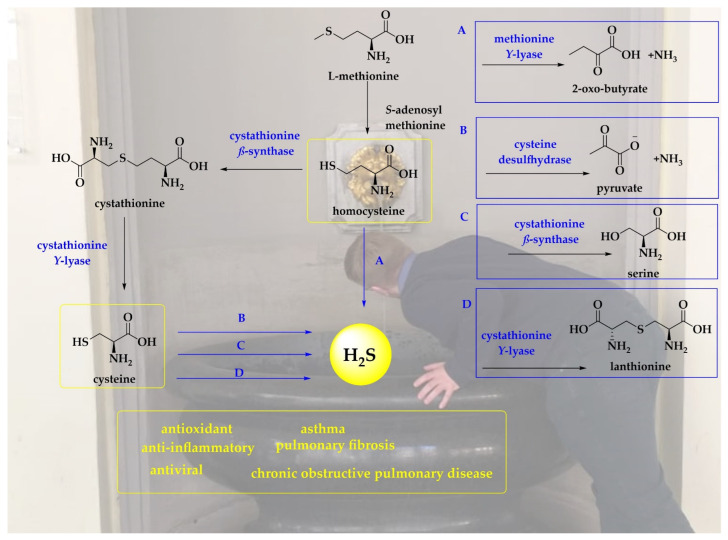
In mammals, H_2_S is produced via several pathways. Cystathionine-*β*-synthase (CBS) and cystathionine-*γ*-lyase (CSE) interact with cysteine to produce H_2_S following the reverse transsulfuration pathway and *α*, *β*-elimination, respectively [39,40]. The production of H_2_S also involves enzymes such as methionine-*γ*-lyase and cysteine desulfhydrase [24]. The scheme is superimposed on a photo of the Elisenbrunnen in the German city of Aachen, a popular source of sulfur-rich water drunk frequently by the local population (Photo credit to Muhammad Jawad Nasim) [41].

Indeed, H_2_S is no stranger to the human body, also in the hypothetical absence of bacteria. Human erythrocytes have been reported to generate H_2_S from both organic and inorganic polysulfides at constant concentrations reaching up to 170 µmol (L cells)^−1^ min^−1^ [34,35,36]. The conversion of garlic-derived polysulfides to H_2_S involve thiol- and glutathione-dependent reactions supported by glucose [36]. Moreover, the interactions of GSH or nicotinamide adenine dinucleotide (NADH) or nicotinamide adenine dinucleotide phosphate (NADPH) in the cell lysate also produce H_2_S [34,35,36]. The conversion of cysteine to cysteine per- and -polysulfides is catalyzed by cysteinyl tRNA synthetase (CARS2). Thioredoxin and GSH may reduce cysteine per- and -polysulfides employing NADPH as a source of electrons to produce H_2_S [37]. The non-enzymatic pathways involve the catalytic production of H_2_S from cysteine by iron and Vitamin B_6_ under physiological conditions [38]. Other major pathways for the production of H_2_S in animals and humans are presented in Figure 1.

H_2_S plays a role as a signaling molecule, similar to other gaseous transmitters, such as nitric oxide (^●^NO) and carbon monoxide (CO). Among these molecules, H_2_S is rather special as it is able to dissociate to hydrogen sulfide ions (HS^−^) with a dissociation constant p*K_a1_* of 7.0, suggesting that at a physiological pH of 7.4, 28% of total hydrogen sulfide exists as H_2_S whilst 72% exists as HS^−^ and very little as S^2−^. Indeed, the high *pK_a2_* value of more than 12 suggests that S^2−^ may not be formed in physiological systems [42]. H_2_S may therefore occur in considerable concentrations of up to 35–80 µM in the human bloodstream compared to 24.4–24.8 µM for ^●^NO [43,44].

Notably, animals and humans are unable to access H_2_S via the reductive avenue employed by bacteria in the notorious cesspits and the gut mentioned already. Therefore, sulfate (SO_4_^2−^) and to a lesser extent sulfite (SO_3_^2−^), which may be consumed directly or indirectly via daily nutrition, are not immediate sources of H_2_S, unless, of course, they are processed by gut bacteria.

As volatile H_2_S is highly soluble in aqueous solutions and in lipids, it is able to cross the plasma membranes and finds its way towards the lungs. Here, it may actually even cure a few respiratory diseases, such as obstructive respiratory disease, emphysema, pulmonary fibrosis, pulmonary inflammation, pancreatic inflammatory/respiratory lung injury, bronchiectasis, and bronchial asthma [45,46]. Intriguingly, serum concentrations of H_2_S vary significantly amongst various respiratory conditions including asthma (comparably low with 55.8 ± 13.6 μM vs. 75.2 ± 13.0 μM in healthy subjects) and chronic obstructive pulmonary disease (comparably high with 149 ± 77.6 μM vs. 90.6 ± 52.7 μM in healthy smokers and 91.0 ± 62.2 μM in healthy non-smokers), highlighting the possible application of H_2_S as a biomarker [47].

Furthermore, the biological activity of H_2_S and its physiological manifestations have recently been linked to the prevention and treatment of COVID-19. H_2_S may (a) block the entry of this virus into cells by interfering with the host receptors, (b) stop the viral replication by interfering with RNA-dependent RNA polymerase (RdRp), and (c) provoke the activation of a hyperinflammatory cytokine storm against the virus [48,49,50,51].

Intriguingly, H_2_S is also produced in the skin by both enzymatic and non-enzymatic pathways. Surprisingly, CSE, CBS, and 3-mercaptopyruvate sulfur transferase which are found in brain have also been detected in the epidermis [52]. The non-enzymatic pathways include reduction of elemental sulfur by GSH involving NADH or NADPH as electron donors or the release of H_2_S from iron–sulfur proteins containing Fe_2_S_2_, Fe_3_S_4_, or Fe_4_S_4_ clusters, such as ferredoxins and Rieske proteins [34,53]. Irrespective of the pathway of production, H_2_S plays several vital physiological roles in skin, such as promotion of cell proliferation, vasodilatation, apoptosis, and inflammation. As in the case of lung disorders, the amounts of H_2_S produced in various dermatological diseases such as psoriasis, melanoma, and other dermatoses vary [52,54].

### 2.2. Sulfur Dioxide (SO_2_)

**Figure 2:**
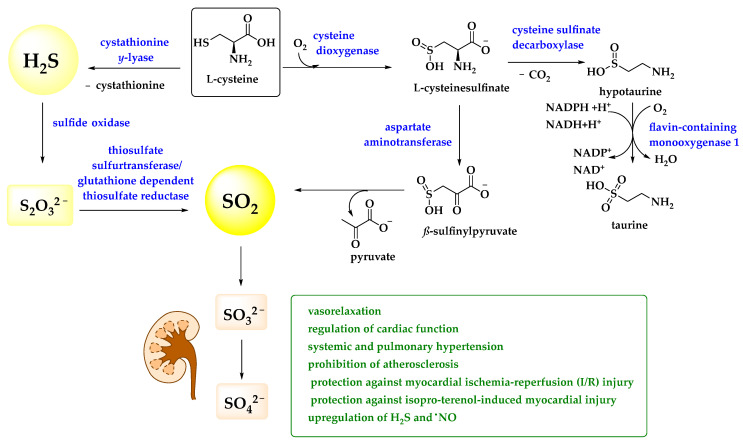
In humans, SO_2_ is produced from L-cysteine and metabolized to SO_3_^2−^ and SO_4_^2−^, which are excreted via the urine. A brief list of biological activities is provided and more details can be found in the text [62,63,64].

Compared to H_2_S, the biological roles of sulfur dioxide are less apparent and prominent. Admittedly, SO_2_ is a rather toxic sulfur compound, an environmental pollutant associated with acidic rain (containing sulfurous acid H_2_SO_3_) and severe damage to forests in the 1970s and 1980s [55]. It is also occasionally used to fumigate hospital wards and as a preservative for nuts and fruits and, of course, wine [56,57]. Nonetheless, Ji et al. have reported that ionic sulfite(s), derivatives of SO_2_, has been found in human serum at a concentration of up to 10 μM whilst Du et al. have detected an endogenous SO_2_ concentration of 15.54 ± 1.68 μM in rat plasma [58,59]. Under physiological conditions, SO_2_ is mostly found as sulfite SO_3_^2−^ ion, as it dissociates in water with first and second p*K_a_* values of 1.81 and 6.97, respectively, suggesting a 1:3 ratio for HSO_3_^−^ and SO_3_^2−^ [60]. Indeed, similar to H_2_S, SO_2_—in the form of (hydrogen) sulfite—may be produced in the gastrointestinal tract by the reductive actions of bacteria, and even inside the human body by the transamination of sulfur-containing amino acids thanks to the activity of aspartate aminotransferase (AAT) (Figure 2) [61].

To date, a brief literature review on possible beneficial roles of SO_2_ in animals and humans only retrieves a few, yet quite interesting publications on a postulated role of the gas and its ions in cellular signaling, notably in the context of pathologies affecting the cardiovascular, nervous, and respiratory systems. In these studies, it looks as if SO_2_ may be involved in the normal physiological functions of the cardiovascular system, including vasorelaxation and regulation of cardiac function [62]. Moreover, SO_2_ may improve both systemic and pulmonary hypertension, prohibit atherosclerosis, and provide protection against myocardial ischemia-reperfusion (I/R) injury and isoproterenol-induced myocardial injury [62]. Intriguingly, SO_2_ is able to upregulate endogenous levels of other gasotransmitters, such as H_2_S or ^●^NO, in various cardiovascular disorders, such as systemic or pulmonary hypertension and atherosclerosis [62].

Mechanistically, endogenous SO_2_ manifests its biological activity in these studies through its involvement in the regulation of apoptosis. In this context, it has been reported that the improvements in the vascular remodeling in hypertension and pulmonary hypertension are achieved through increasing apoptosis of vascular smooth muscle cells (VSMCs) and prohibiting the apoptosis of vascular endothelial cells (ECs) [65]. Endogenous SO_2_ also provides cardio-protection by suppressing the apoptosis of cardiomyocytes [66]. Whether or not such initially beneficial actions are—at closer inspection—really desirable and may even be used in the context of therapy is a major and so far not really answered question.

Likewise, SO_2_ serves as a double-edged sword in the nervous system where on one hand it facilitates kainic acid induced hippocampal neuronal apoptosis leading to epileptic brain damage, whilst on the other hand, it decreases neuronal apoptosis and provides neuro-protection in recurrent febrile seizure and global brain I/R injury [67,68]. Furthermore, endogenous SO_2_ is closely linked with the pathogenesis of blinding retinal disease since the downregulation of endogenous SO_2_ has been reported to result in increased apoptosis of 661w retinal photoreceptor cells [69]. Then again, in the respiratory system, endogenous SO_2_ has been reported to assist the apoptosis of polymorphonuclear neutrophils (PMNs) and to inhibit the apoptosis of alveolar macrophages in order to provide protection against acute lung injury [63,70]. In any case, the relationship between SO_2_ and apoptosis is complicated, often dependent upon its exact concentration and the cell type exposed to it, and clearly requires further investigation.

## 3. Volatile Natural Products

Compared to structurally simple volatile inorganic sulfur compounds such as H_2_S and SO_2_, the field of organic sulfur compounds is considerably more diverse, and many of them are secondary metabolites serving as antioxidants or protectants against infection or predators [71]. The high reactivity often associated with such sulfur compounds then translates into powerful biological activities also outside the plants, bacteria, fungi, or animals of origin. Nonetheless, there are also quite a few limitations when it comes to volatile organic sulfur molecules. In general, these molecules need to be produced, stored, and/or distributed by the producer in a more or less controlled and stable manner, which is a challenge for any plant, bacterium, fungus, or animal. Secondly, as some of these molecules are rather active in biology, they should also not necessarily (re-)act prematurely, for instance by attacking the producer rather than the target. Furthermore, their distribution needs to be controlled, which is a clear challenge for a gas unless there are (de-)protonated ionic forms circumventing this issue. Fourthly, most of these molecules can be metabolized inside the target organism and one may therefore need to pay more attention to the more durable metabolites rather than the original volatile molecule itself.

### 3.1. Metabolites of H_2_S

H_2_S has the tendency to oxidize into inorganic H_2_S_x_ species with x ranging from 1–7 before further oxidation which produces elemental S_8_ and a precipitate [72,73]. The exact number of sulfur atoms present in H_2_S_x_ species under physiological conditions remains a matter of thorough investigation and may not only occur by spontaneous, random oxidation. There is speculation, for instance, that the production of H_2_S_x_ species could involve either the enzyme 3-mercaptopyruvate sulfurtransferase (3MST) and also interactions of H_2_S with ^●^NO. In contrast to H_2_S, which *S*-thiolates oxidized cysteine residues and reduces cysteine disulfide bonds, H_2_S_x_ can form per- and polysulfide motifs attached to cysteine residues of target proteins, thus modifying their activity [74]. Not surprisingly, biological activities of H_2_S_x_ include, among others, regulation of the activity of the tumor suppressor Phosphatase and Tensin Homolog deleted on Chromosome 10 (PTEN), facilitation of the translocation of Nuclear factor-erythroid factor 2-related factor 2 (Nrf2), and suppression of the activity of Glyceraldehyde 3-phosphate dehydrogenase (GAPDH) [75,76,77].

In mammalian mitochondria, the conversion of H_2_S to the polysulfide is catalyzed by sulfide quinone oxidoreductase (SQR), and this polysulfide is subsequently transferred to sulfite to produce thiosulfate (S_2_O_3_^2−^) or GSH persulfide (GSSH) [78,79]. Similarly, sulfide-producing enzymes, such as CSE and CBS are able to catalyze the *beta*-elimination reaction of cystine, to produce cysteine persulfide and polysulfide [80,81,82]. Sulfenic acids (RSOH) and *S*-nitrosothiols (RSNO) are also two very important players for the non-enzymatic production of persulfide. Protein thiols are subject to reversible oxidation to sulfenic acid by *S*-sulfenylation [83]. The pre-existing *S*-sulfenylation has been proposed to be responsible for the persulfidation of GAPDH and bovine serum albumin (BSA) [84,85]. *S*-nitrosothiol represents another form of oxidized thiol, which reacts with H_2_S to produce persulfides [86]. In contrast, sulfhydral radicals (^●^SH) are produced as a result of oxidation of H_2_S by metal centers, such as copper and iron, and may then react with free thiols and H_2_S to produce protein persulfides and polysulfides, respectively [87]. 

Besides oxidized metabolites of H_2_S, we should therefore turn our immediate attention to the metabolites of H_2_S, which is frequently methylated in animals and humans and therefore comes in concert with molecules such as methyl sulfide (MS, CH_3_SH), dimethyl sulfide (DMS, CH_3_SCH_3_), and the trimethyl sulfonium ion ((CH_3_)_3_S^+^) [27,88]. The trimethyl sulfonium ion is water-soluble and excreted via the kidneys. MS and DMS are volatile with boiling points of 6 °C and 37 °C, respectively. They circulate the human body and are released via the lung and skin. Concentrations of up to 178 ppb (3.78 µM) for MS and 35 ppb (0.56 µM) for DMS have been observed in the breath and therefore may induce some biological activities in the cells lining the airways [89].

Apart from that, the literature is vague on the biological impact of MS and DMS, especially at lower (micromolar) concentrations and prolonged exposure. Among the few studies addressing the biological activities of such metabolites, there are reports that the presence of VSCs, including the methylated metabolites of hydrogen sulfide, may be associated with chronic periodontitis and other oral conditions [90,91]. Then again, DMS has also been reported to provide an effective shield against oxidative stress and even to prolong the lifespan of mammals through a catalytic mechanism involving methionine sulfoxide reductase A [92].

### 3.2. Simple Alkyl Sulfides

Compounds such as dimethyl sulfide (CH_3_SCH_3_) and diethyl sulfide (C_2_H_5_SC_2_H_5_) may indeed not be the most active sulfides in biology, as the dialkyl monosulfide motif tends to be rather unreactive under physiological conditions. It is also present, for instance, in the proteinogenic amino acid methionine, and although its sulfur can be oxidized to a sulfoxide and theoretically also to a sulfone, this is a rare event and various methionine *S*-oxide reductases are at hand to reverse this modification [93].

Moving from the mono- to the di-, tri-, or tetrasulfide changes this reactivity considerably [94]. Unlike the carbon-sulfur bond, the sulfur-sulfur bond is highly reactive. Besides oxidation of the sulfur to an *S*-oxide, in this case a thiosulfinate (RS(O)SR’) or thiosulfonate (RS(O)_2_SR’), it also allows reduction, for instance via an eloquent thiol/disulfide exchange mechanism [95,96]. Not surprisingly, the dialklyl disulfides and dialkyl polysulfides (RSxR’, x ≥ 2) also exhibit a range of pronounced biological activities, for instance as antioxidants and antimicrobial agents [97,98]. Among them, the dipropyl sulfides occur naturally in onions and show a palette of activities, mostly cytotoxic and antimicrobial, and mostly due to their ability to oxidatively modify certain target proteins and enzymes [99]. As a rule of thumb, the longer the sulfur-sulfur chain, the higher the reactivity and also the biological activity, with the caveat that longer sulfur-sulfur chains are also becoming more unstable. Most studies therefore consider the tri- and tetrasulfides as the most promising compounds for biological applications.

To underline the importance of this class of simple yet extraordinarily active compounds, we shall briefly mention dimethyl disulfide (DMDS, CH_3_SSCH_3_, melting point −85 °C, boiling point 110 °C) and dipropyl trisulfide (DPTS, C_3_H_7_SSSC_3_H_7_, boiling point 72°C) as prominent and perhaps also representative substances in this field. The naturally occurring DMDS shows antifungal activity against *Sclerotinia minor* by attacking the membrane of this fungus and also induces systemic resistance against white mold in host plants [100]. This simple disulfide also inhibits the two plant pathogens *Agrobacterium tumefaciens* and *Agrobacterium vitis* by functioning as a bacteriostatic agent [101,102]. Although DMDS has a boiling point of 110 °C, it has been applied air bound and, perhaps not surprisingly, is in use already as an innovative fumigant under the trade name PALADIN^®^ to target soil-borne plant pathogens [103]. There are also studies indicating that DMDS may interfere with bacterial quorum sensing communication by significantly suppressing the transcription of *N*-acyl homoserine lactone synthase genes [102,104]. In any case, this and other simple volatile disulfides possess considerable potential for practical applications in the—literally—field of antimicrobial fumigation.

Dipropyl tetrasulfide (DPTTS, C_3_H_7_SSSSC_3_H_7_, boiling point 127 °C), on the other hand, is less volatile and has been studied extensively as a redox modulator in many disease models, among them a mouse model indicative of systemic sclerosis (scleroderma) [99]. In this model, DPTTS derived synthetically and also isolated from onions shows considerable beneficial properties by selectively killing the diseased fibroblasts and, thereby, counteracting this disease. When exposed to DPTTS, the concentration of H_2_O_2_ significantly increases in HOCl-fibroblasts as compared to normal fibroblasts. Moreover, DPTTS provides higher cytotoxic and pro-apoptotic activities in HOCl fibroblasts than in normal fibroblasts [99]. Once again, the mechanisms underlying this activity may be complex, and in any case related to the pronounced redox activity associated with the sulfur-sulfur bond and in need of further investigation.

**Figure 3:**
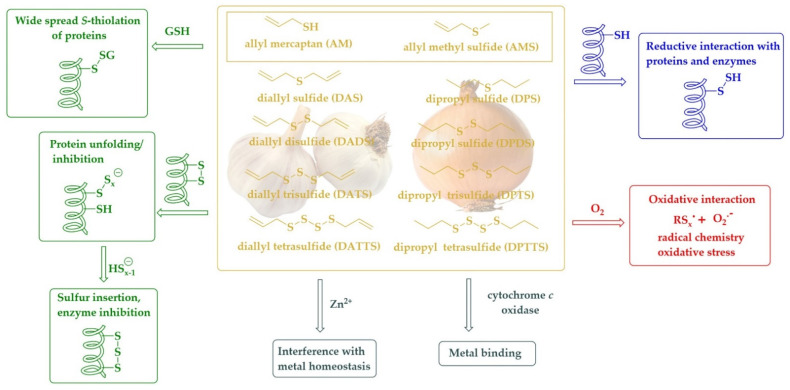
Allyl and propyl mono- and polysulfides found in garlic and onion may serve as multifunctional agents able to interact with thiol residues of proteins and enzymes, interfere with metal homeostasis, bind to metals, and induce oxidative stress via oxidative interactions.

The discussion of DPTTS from onions almost immediately also brings us to its relative in garlic, diallyl trisulfide (C_3_H_5_SSSC_3_H_5_, DATS). Indeed, DATS is equally, if not more active biologically compared to DPTTS and is also the source of centuries of medicinal inspiration and a plethora of publications [105,106,107]. Similar to DPTTS, DATS is volatile with a boiling point of 120 °C, and there are numerous anecdotes of DATS and its relatives diallyl disulfide (C_3_H_5_SSC_3_H_5_, DADS, boiling point 187.5 °C) and diallyl tetrasulfide (C_3_H_5_SSSSC_3_H_5_, DATTS, boiling point 45 °C) fuming out entire incubators when applied in Petri dish-based studies [11]. The biological activities associated with these diallyl sulfides range from antioxidant and protective to redox modulation and cytotoxic and are usually associated with the reactivity of the sulfur-sulfur bond acting in cahoots with the double bond of the allyl group. A brief overview of such compounds and their activities have been depicted in Figure 3. As mentioned already, the monosulfides, namely dipropyl sulfide (C_3_H_7_SC_3_H_7_, DPS, boiling point 142.9 °C) and diallyl sulfide (C_3_H_5_SC_3_H_5_, DAS, boiling point 138.6 °C) are also found in onions and garlic, respectively, although the biological activities associated with these less reactive compounds is also more restricted. DAS, for instance, has been reported to serve as a selective inhibitor of cytochrome P450 2E1 (CYP2E1), and therefore it is not only able to inhibit the cellular toxicities associated with alcohol and xenobiotic drugs; it also limits the cellular toxicities associated with HIV proteins and diabetes [108].

As for practical applications, the polysulfides from onions and garlic are employed already in the field of agriculture, in essence in the form of sprays and pellets [109]. To the best of our information, their possible use in fumigation has not been explored further so far. Notably, minute amounts of diallyl sulfides are present in so-called garlic breath after consumption of raw garlic [110]. The concentrations of DAS, DADS, and DATS increase from around 6 ppb (52.5 nM), 10 ppb (80.5 nM), and 3 ppb (16.8 nM) to 13 ppb (113.8 nM), 130 ppb (1.1 µM), and 5.5 ppb (30.9 nM), respectively, in the breath exhaled after ingestion of raw garlic [111]. Intriguingly, the concentration of DADS reaches its peak of 130 ppb in around two hours after ingestion of garlic followed by its rapid removal. The concentration of dimethylsulfide (DMS), in contrast, increases from 30 ppb (482.9 nM) to 60 ppb (965.7 nM) in 8 h and reaches the peak of 90 ppb (1.5 µM) in around 24 h after ingestion of garlic, possibly formed via the H_2_S pathway discussed already [111]. If and to which extent such low and sub-micromolar concentrations of DAS, DADS, and DATS in breath may exert any notable biological activity on cells lining the respiratory system is so far unclear, although not entirely impossible.

Indeed, such sulfur compounds are also excreted via the skin in the form of “human skin gas” and may therefore not only affect the respiratory system, but also the skin cells they encounter during their release. Sato et al. have recently reported that DADS presents a mean emission flux of 0.18 ng (1.23 pmol) cm^−2^ h^−1^ (n = 30) from the surface of human skin without ingestion of garlic. The consumption of 45 g of grilled garlic results in increased emission flux of DADS achieving a maximum of 4.3–5.2 ng (29.39–35.55 pmol) cm^−2^ h^−1^. Even after 8 h, the values of emission fluxes are higher than the base-line values [112]. As for breath, whether such small, albeit sustained, concentrations of these substances exert any major biological effects in and/or near the skin requires further investigations. It may, for instance, be feasible that some of these substances accumulate or enrich in the lung and skin cells and subsequently provide an antioxidant shield or affect microbes attached to these organs. In any case, the topic of exhalation of intact or metabolized sulfur compounds is rather stimulating as it brings the (active) sulfur compounds into intimate contact with tissues where they may have numerous benefits.

### 3.3. Metabolites

The discussion of exhaled rather than inhaled volatile and biologically active molecules essentially brings us to the various metabolites of natural sulfur compounds. In this context, it is essential to note that the metabolites, and not necessarily the original sulfur compounds, may themselves be volatile and/or active, as is the case, for instance, for several ingredients found in the *Allium* plants, such as alliin (*S*-allyl-L-cysteine) [8]. Equally notably, such metabolites are often less reactive than the original compounds as they have already lost some of their (re-)activity inside the body. Thus, the exhaled metabolites tend to have a different and generally lower biological activity when compared to the original substance taken up orally [113].

In the case of the infamous “garlic breath”, the simple alkyl sulfides discussed in Section 3.2, e.g., DMS, DAS, DADS, and DATS are still present, albeit in rather low concentrations [111]. This is partially due to release of some of these substances from the mouth and stomach, in essence before they have been taken up and metabolized. Excretion through the skin and lung differs, as mentioned already, and here one finds a different spectrum of—metabolized—volatile sulfur compounds, namely allyl methyl sulfide (C_3_H_5_SCH_3_, AMS, boiling point 92 °C).

**Figure 4:**
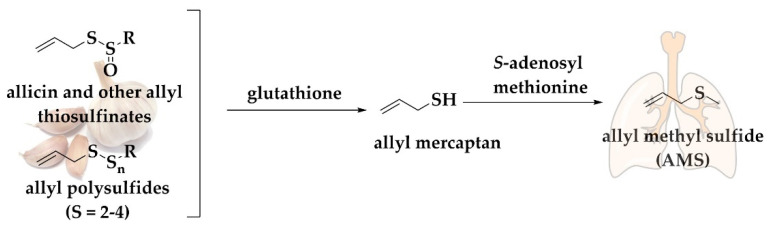
Generic pathway for the conversion of allyl thiosulfinates and polysulfanes to AMS. Allyl polysulfides are metabolized mainly to allyl methyl sulfide with allyl mercaptan as an intermediate, see text for details.

Allicin is converted under physiological conditions to polysulfides and then to this volatile monosulfide, AMS, which is present as a major constituent of garlic breath (Figure 4). Studies have shown that after the consumption of raw garlic, the content of AMS in breath is linearly proportional to the amount of allicin consumed [114]. AMS represents the main metabolite of allicin exerted via the breath. Generally, 90% of the allicin consumed is turned into AMS, whilst allyl mercaptan (C_3_H_5_SH) is only a temporary intermediate in the formation of AMS. Allicin-derived DADS and DATS are also metabolized mainly to AMS. Furthermore, AMS is an active metabolite, responsible for the ability of allicin to increase the acetone levels in breath. Intriguingly, alveolar breath has been shown to contain 18% higher AMS concentrations compared to whole breath [115]. The release of AMS is not only confined to lungs since it has been recently reported that AMS presents a mean emission flux of 0.22 ng (2.50 pmol) cm^−2^ h^−1^ (n = 30) from the surface of human skin without ingestion of garlic. The consumption of 45 g of grilled garlic results in increased emission flux of AMS achieving a maximum of 2.8–3.0 ng (31.76–34.03 pmol) cm^−2^ h^−1^ [112].

AMS has been associated with several potential biological applications, especially in the treatment of lung cancer [116]. AMS, alongside DADS and DAS, has been reported to reduce the formation of cancerous lung nodules by 54.8% in the C57BL/6 mice model [116]. Furthermore, a recent study shows that AMS may act as an efficient antiviral agent. Based on a computational study, AMS may attach to the phospholipid surface of a virus, such as the SARS-CoV-2 virus, and thereby denature the virus [117]. Another study suggests that AMS might also be useful as a fumigation agent. Here, AMS shows a significant toxicity against *Actinobacillus pleuropneumoniae*, which causes Pleuropneumonia, a serious lung disease in pigs [118]. AMS may also be beneficial in the treatment of Alzheimer Disease (AD). In this case, 500 µM AMS inhibit *β*-secretase, acetyl choline esterase, and butyrylcholinesterase by 18.4%, 24.5%, and 21.0%, respectively [119].

### 3.4. The Durian Fruit—A Treasure Chest of Public Annoyance and Volatile Sulfur Compounds

**Figure 5:**
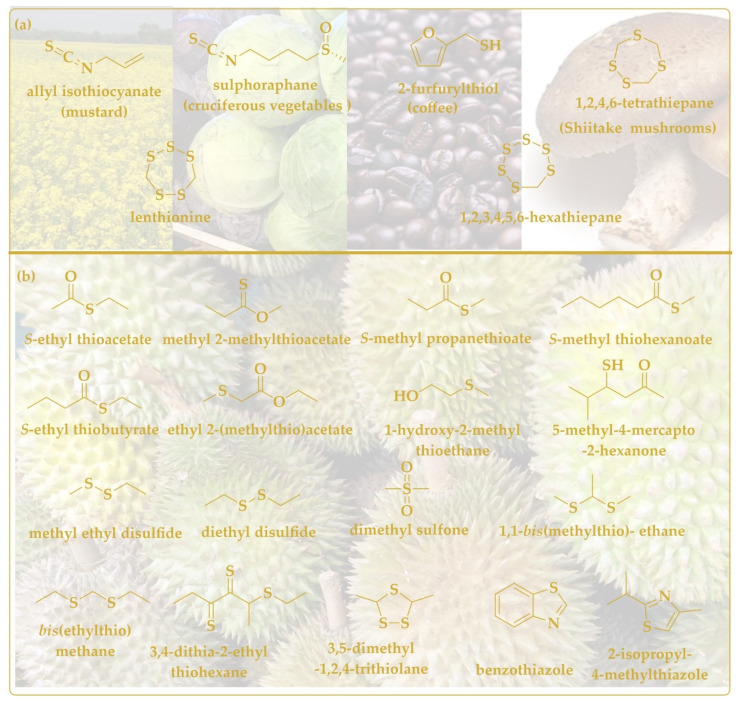
A brief overview of volatile sulfur species found in natural products such as mustard, cruciferous vegetables, coffee, and shiitake mushroom (**a**), and in durian fruit (**b**).

Onions and garlic are not the sole producers of biologically active sulfur compounds. Broccoli has been mentioned already and mustard must also be on the rollcall, as these vegetables contain biologically active sulforaphane and allyl isothiocyanate, respectively (Figure 5a). Indeed, the less volatile sulforaphane (boiling point 368.2 °C) is often associated with antioxidant and preventive activities, whereas the more volatile mustard oil (boiling point 170 °C) shows pronounced anticancer, antioxidant, and antimicrobial activities in various in vitro and cell culture studies [120,121].

Compared to these popular culinary plants present in almost every cuisine across the globe, the durian fruit cultivated in Southeast Asia is more for lovers of extreme tastes [122]. Its pungent smell is often a matter of considerable annoyance and its transport and public consumption is frequently forbidden [123].

The durian fruit is rich in sugars, fat, fiber, and micronutrients, such as potassium. It is also home to a range of rather smelly volatile compounds, such as esters, sulfur, thioacetals, thioesters, thiolanes, and alcohols, some of them pictured in Figure 5b [124]. Thanks to the presence of these substances, the durian fruit is cherished in some quarters for its supposed anti-hyperglycemic, anti-atherosclerotic, probiotic and even anti-proliferative activities and its individual sulfur-containing ingredients lend themselves for further investigations [125].

## 4. Conclusions

Our brief encounter with volatile, biologically active sulfur agents has shown that quite a palette of such substances is present as natural products in plants, fungi, animals, and microbes. Many of these compounds serve as antioxidants or as part of host defense and therefore may also be of interest for potential uses in the fields of agriculture and medicine. Among the inorganic substances, H_2_S clearly stands out as an exogenous toxic gas at higher concentrations and an endogenous signaling molecule in humans and animals at lower concentrations. If and how its oxidized companion SO_2_ may also serve as a signaling molecule is still a matter of research and debate. More eloquent sulfur-containing agents are found in plants such as garlic and onions, and besides possible uses as culinary delights may also produce metabolites during (in-)digestion, which themselves may be active biologically, inside the body and during excretion. As usual, reactivity and activity in vitro alone are not sufficient, and concentrations—and periods of exposure—also matter if one tries to turn such sulfur compounds into good antioxidants or antimicrobial agents. The field of volatile sulfur compounds, especially the ones of natural origin, is therefore one worth considering and cultivating, as such compounds may have rather valuable applications in agriculture, medicine, and also as a culinary highlight in our daily nutrition. So, let’s go research, let’s go.
